# Eosinophilic coronary periarteritis causing recurrent coronary spasms

**DOI:** 10.1007/s12471-025-01932-9

**Published:** 2025-02-20

**Authors:** Manon Graman, Albertus Josephus Voogel

**Affiliations:** 1 Emergency Care, OLVG, Amsterdam, The Netherlands; 2 Department of Cardiology, Spaarne Gasthuis, Haarlem, The Netherlands

**Fig. 1:**
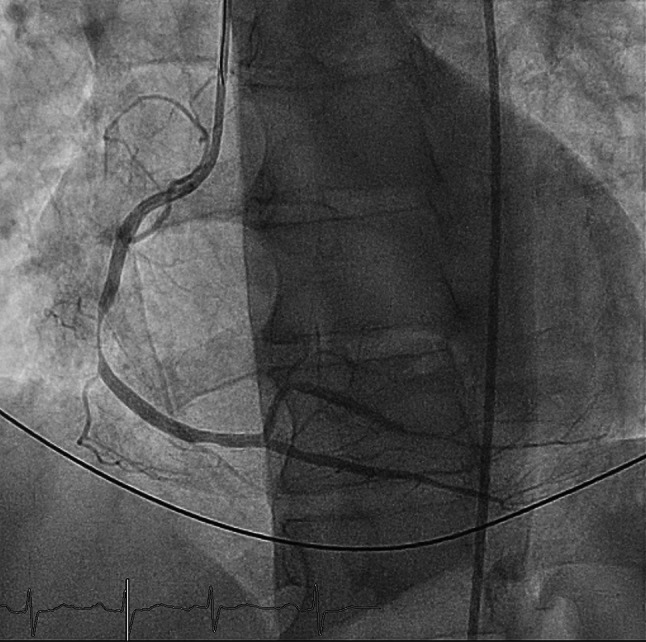
Angiography of coronary spasms in RCA

Eosinophilic coronary periarteritis (ECPA) is a rare cause of coronary vasculitis. Most reports describe autopsies of patients with sudden cardiac death and a history of vasospastic angina [1–3]. We report a case of a previously healthy 59-year old male with ventricular fibrillation directly following surgical resection of atopic nasal polyps. Invasive coronary angiography showed subtotal stenoses of the RPL and RCA (Fig. 1), which were treated by stenting. The patient was discharged with antiplatelet and spasmolytic therapy, although already suspecting ECPA. Within 6 months patient presented with recurrent chest pain and invasive angiography revealed coronary spasms in different coronary territories. Laboratory testing showed elevated eosinophilic granulocytes (0.7 × 10^9^/L), which further substantiated the suspicion of ECPA. High dose prednisolone was started, which resulted in normalisation of the eosinophil count and improvement of angina complaints. ECPA should be considered in patients with angina lacking traditional risk factors. Definite diagnosis can only be confirmed through PA.
